# Altered tryptophan metabolism as a contributor to cognitive impairment in chronic kidney disease: a narrative review

**DOI:** 10.3389/fnins.2026.1872778

**Published:** 2026-07-17

**Authors:** Harish Selvaraj, Varadharajan Jayaprakash, Janardanan Subramonia Kumar, Gerry George Mathew, Karthikeyan Sundaram, Venkataraman Prabhu

**Affiliations:** 1 Division of Medical Research, SRM Medical College Hospital and Research Centre, SRMIST, Chengalpattu, Tamil Nadu, India; 2 Department of Nephrology, SRM Medical College Hospital and Research Centre, SRMIST, Chengalpattu, Tamil Nadu, India; 3 Department of General Medicine, SRM Medical College Hospital and Research Centre, SRMIST, Chengalpattu, Tamil Nadu, India; 4 Department of Herbal Pharmacology and Environmental Sustainability, Chettinad Hospital and Research Institute, Chettinad Academy of Research and Education, Kelambakkam, Tamil Nadu, India

**Keywords:** chronic kidney disease, cognitive impairment, tryptophan, kynurenine, gut microbiome, serotonin, melatonin

## Abstract

Approximately 40% of patients with chronic kidney disease (CKD) experience cognitive impairment (CI), which is strongly associated with increased mortality. CI is driven by multiple factors, including vascular injury, accumulation of uremic toxins, disruption of the blood–brain barrier, and chronic inflammation. Recent evidence suggests that kidney disease and neurocognitive decline are mechanistically linked through dysregulated tryptophan metabolism. Tryptophan is metabolised through three main pathways: the kynurenine, indole, and serotonin pathways, each producing bioactive metabolites with distinct neurophysiological effects. The hallmarks of CKD include chronic inflammation, gut microbial dysbiosis, and impaired renal clearance, all of which alter tryptophan metabolism. Inflammation drives tryptophan metabolism towards the kynurenine pathway, increasing the formation of neurotoxic compounds that promote oxidative stress, excitotoxicity, and neuronal injury. However, reduced availability of tryptophan for serotonin synthesis impairs serotonergic signalling and neurotransmission, as well as melatonin biosynthesis, thereby contributing to circadian rhythm disturbances and impaired glymphatic clearance. Concurrently, gut dysbiosis and reduced renal clearance promote the accumulation of indole-derived uremic toxins, leading to endothelial dysfunction, neuroinflammation, and disruption of the blood–brain barrier. This review highlights the current evidence of dysregulated tryptophan metabolism in CKD and its impact on the pathogenesis of neurocognitive complications. The review also discusses potential biomarkers and therapeutic strategies, including kynurenine pathway inhibitors, gut microbiota modulation, uremic toxin adsorption, melatonin supplementation and personalised medicine to mitigate cognitive impairment in CKD.

## 1 Introduction

Approximately 850 million people worldwide are living with kidney diseases, which encompass both acute and chronic kidney disease (CKD). Of these, more than 700 million have CKD, which has become one of the most common morbidities and a leading cause of mortality worldwide. It is a significant and growing global health concern. Due to CKD, approximately 3.6 million people died globally in 2019 (Jager et al., 2019; Francis et al., 2024). India and China together account for more than 30% of all cases globally, with India alone contributing approximately 115 million affected individuals (Bikbov et al., 2020). The two major root causes of CKD are diabetes mellitus and hypertension. Roughly one in three people with diabetes and one in five people with hypertension will develop CKD (Qureshi et al., 2025). Patients with CKD are at higher risk for numerous systemic consequences, such as anaemia, cardiovascular disease, and bone mineral abnormalities. Beyond these complications, CKD presents a significant association with Cognitive impairment (CI). The causes of developing CI in CKD are multifactorial, including vascular disease, uraemic toxins, blood–brain barrier damage, and metabolic and endocrine changes (Bolignano et al., 2025).

CI is a collection of deficits that affect memory, attention, executive function, visuospatial skills, language, and learning (Viggiano et al., 2020). These impairments collectively reflect disruption in brain function and neurovascular health. This neurological burden in CKD strongly correlates with declining estimated glomerular filtration rate (eGFR), longer dialysis duration, and systemic inflammation, indicating a multifactorial pathogenesis. According to a recent meta-analysis, 40% of patients with CKD developed CI. Among patients undergoing haemodialysis and peritoneal dialysis, the prevalence is higher (53 and 39%, respectively) compared to non-dialysis groups (Zhang et al., 2024). Based on current literature, focusing on neurological complications in CKD patients is essential due to their association with increased mortality, frequent underdiagnosis in routine care, under-recognition in early stages and younger patients, and their significant impact on functional capacity and quality of life (Murtaza and Dasgupta, 2021; Pépin et al., 2023; Xie et al., 2022).

Recent studies have shown that dysregulation in amino acid metabolism is associated with CKD. Altered pathways involving branched-chain amino acids, glutamine, taurine, sulfur amino acids, and tryptophan have been associated with metabolic imbalance, immune dysregulation, and disease progression (Heneberg and Heneberg Šimčíková, 2026). Among these, tryptophan (TRP) metabolism has gained more attention because it interacts with multiple pathogenic processes simultaneously. Emerging evidence suggests that TRP metabolism acts as a multi-organ signalling network that mediates communication between the immune system, brain, liver, kidney, and gut microbiota. TRP-derived metabolites regulate immune responses, inflammation, vascular homeostasis, and tissue function via the aryl hydrocarbon receptor (AhR) and pregnane X receptor (PXR) signalling pathways (Tang et al., 2025).

The mechanism underlying CI in CKD is complex and multifactorial, influenced by established factors such as vascular disease, blood–brain barrier (BBB) dysfunction, anaemia, dialysis-related hemodynamic instability, and systemic inflammation (Xie et al., 2022). Hallmarks of CKD, such as inflammatory activation, gut microbial dysbiosis, and decreased renal clearance, profoundly alter TRP metabolism. This leads to the accumulation of harmful metabolites implicated in oxidative stress, endothelial dysfunction, BBB impairment, neuroinflammation, and neurotransmitter imbalance. Consequently, dysregulation of TRP metabolism is a critical component of the gut-kidney-brain axis and a potential molecular link driving these extrarenal complications. Despite the increasing recognition of TRP metabolism within the gut-kidney-brain axis, several important questions remain unresolved (Tang et al., 2025).

Current evidence is fragmented into kynurenine, indole, and serotonergic pathways, which are often investigated independently despite their close metabolic and functional interconnections. Consequently, the roles of TRP-derived metabolites as mechanistic mediators, diagnostic biomarkers, and therapeutic targets in CKD-associated CI remain incompletely understood, and their clinical translation remains challenging. This review provides a comprehensive overview of dysregulated TRP metabolism within the gut-kidney-brain axis and discusses its mechanistic implications, diagnostic potential, and emerging therapeutic strategies to mitigate CI in CKD (Badawy, 2017).

### 1.1 Overview of tryptophan metabolism

Tryptophan is an essential aromatic amino acid characterised by an indole structure (Palego et al., 2016). In addition to its role in protein synthesis, TRP is crucial for several physiological processes, including Immune regulation, neurotransmitter production, gut microbiota maintances, melatonin biosynthesis, and Nicotinamide Adenine Dinucleotide (NAD+) biosynthesis (Lu et al., 2021). The majority of free TRP undergoes catabolism through three major metabolic pathways: the Kynurenine, Serotonin/melatonin, and Indole pathways. These pathways result in metabolites with unique biological properties that affect immunological, metabolic and neurological functions. An overview of each pathway is given below to facilitate understanding of its potential relevance to CKD-associated CI.

#### 1.1.1 Kynurenine pathway

Under physiological conditions, more than 90% of dietary TRP is metabolised through the KP, primarily in hepatocytes, whereas extrahepatic KP degradation is limited (approximately 5–10%). However, extrahepatic metabolism becomes more significant under immunological conditions or in the presence of chronic inflammation (Badawy, 2017).

**Figure 1:**
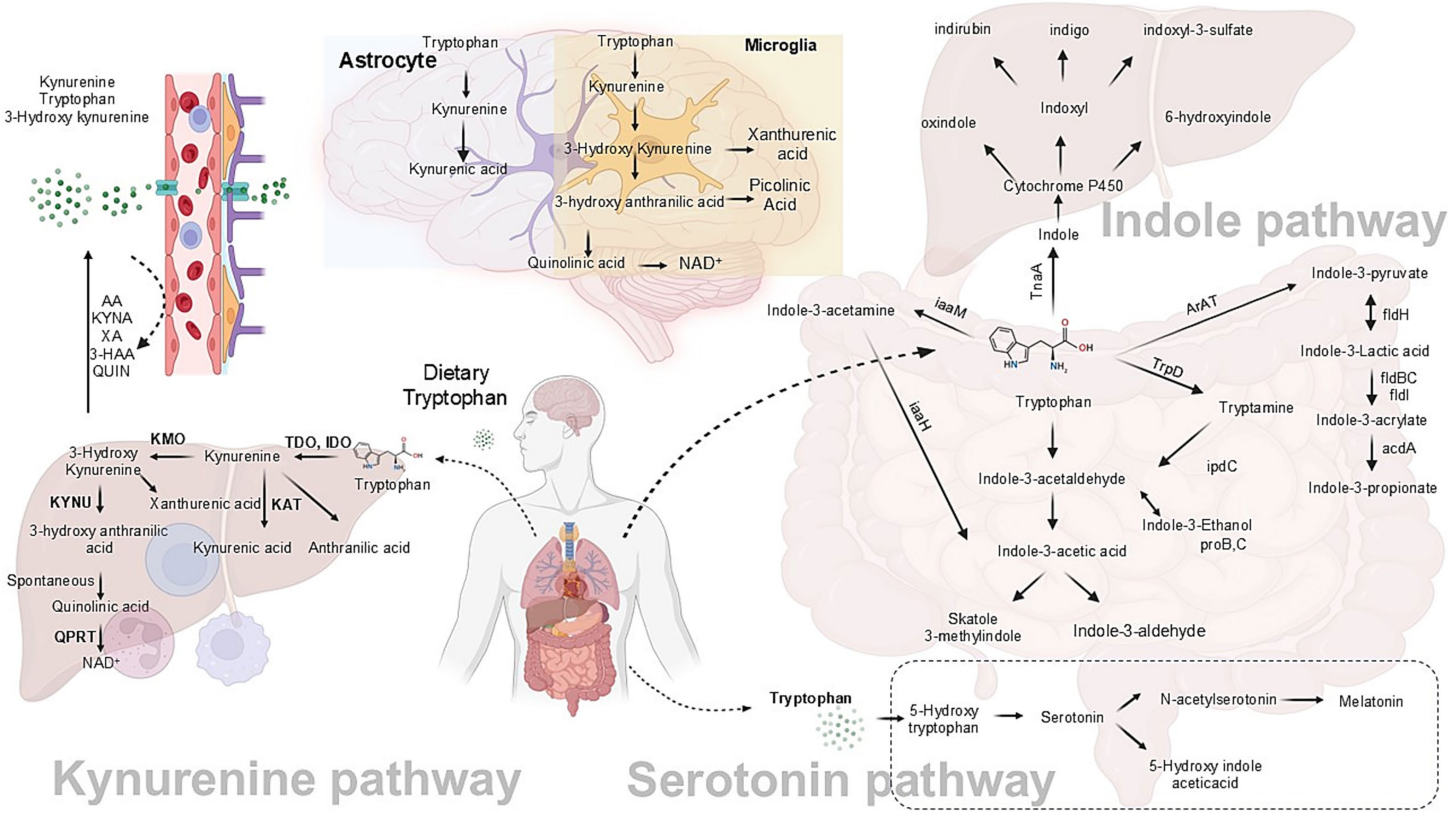
Integrated overview of tryptophan metabolism through kynurenine and serotonin pathways in peripheral tissues and the brain. Dietary tryptophan is metabolised primarily through three pathways: kynurenine, serotonin, and indole pathways. In the kynurenine pathway, tryptophan is converted by either tryptophan 2,3-dioxygenase or indoleamine 2,3-dioxygenase into kynurenine and its downstream metabolites, such as 3-hydroxykynurenine, kynurenic acid, and ultimately nicotinamide adenine dinucleotide (NAD+). These kynurenine metabolites can cross the blood–brain barrier, where they are metabolized differently in astrocytes and microglia, potentially leading to neuroprotective or neurotoxic outcomes. In the gut, tryptophan is metabolised by microbiota via the indole pathway into various derivatives like indole-3-acetic acid and tryptamine, influencing systemic and neurovascular homeostasis and host-microbiota signaling. Additionally, in the serotonin pathway, tryptophan is converted into 5-hydroxytryptophan and serotonin, which can further be metabolised into 5-hydroxyindoleacetic acid and melatonin. These interconnected pathways regulate immunological, metabolic, microbiological, and neuroactive processes crucial for kidney-brain communication. TDO, tryptophan 2,3-dioxygenase; IDO1, indoleamine 2,3-dioxygenase 1; KMO, kynurenine 3-monooxygenase; KYNU, kynureninase; KAT, kynurenine aminotransferase; QPRT, quinolinate phosphoribosyltransferase; TPH, tryptophan hydroxylase; AANAT, arylalkylamine N-acetyltransferase; ASMT, acetylserotonin O-methyltransferase; TnaA, tryptophanase; ArAT, aromatic amino acid aminotransferase; TDC, tryptophan decarboxylase; ipdC, indole-3-pyruvate decarboxylase; iaaM, indole-3-acetamide monooxygenase; iaaH, indole-3-acetamide hydrolase; acdA, acryloyl-CoA dehydratase.

This metabolic cascade is initiated by two distinct cytosolic enzymes, including tryptophan 2,3 dioxygenase (TDO), which is mainly expressed in hepatocytes and regulated by glucocorticoids, and Indoleamine 2,3 dioxygenase (IDO), which is widely distributed in extrahepatic tissues and strongly induced by pro-inflammatory cytokines (Wang Y. et al., 2025). Both enzymes catalyse the conversion of TRP to kynurenine (KYN). Consequently, systemic inflammatory conditions favour increased flux through the KP, driving the enhanced production of downstream neuroactive kynurenine metabolites. Within the central nervous system, KYN is metabolised via two different branches. In the neuroprotective branch, astrocytes utilise kynurenine aminotransferases (KATs) to convert KYN into kynurenic acid (KYNA) (Liu et al., 2018). Conversely, in the neurotoxic branch, microglia process the KYN into 3-hydroxykynurinine (3-HK) by Kynurenine 3-monooxygenase (KMO), KMO is highly active in the liver and kidneys but minimally expressed in the healthy brain. 3-HK induces neurotoxicity by generating reactive oxygen species (ROS), leading to oxidative stress and mitochondrial damage (Figure 1) 3-HK is subsequently hydrolysed by Kynureninase to form 3-hydroxyanthranilic acid (3-HAA) which can act as a pro-oxidant at higher concentrations, especially in inflammatory states. Finally, 3-HAA is converted into quinolinic acid (QA) and hence ultimately produces NAD+ (Mor et al., 2021). The balance between neurotoxic and neuroprotective pathways is evident in the physiological state, as it involves the interplay among microglia, astrocytes, and neurons. The kynurenine pathway in astrocytes has a dual role. Under normal conditions, it is neuroprotective and produces NAD+, which improves cellular energy status. In disease conditions, there will be dysregulation in TRP metabolism, especially in the Kynurenine pathway (Ostapiuk and Urbanska, 2022).

#### 1.1.2 Serotonin pathway

Serotonin is important neurotransmitter; around 95% of serotonin is synthesised in the enterochromaffin cells of the intestinal mucosa, which is stored in platelets. A small amount is formed in serotonergic neurons in the raphe nuclei of the brainstem, which regulates mood, perception, memory, and stress responses (Kanova and Kohout, 2021).

TRP is the major precursor for serotonin; it’s synthesised in just two steps. In the very first step, TRP is hydroxylated into 5-hydroxytryptophan by the tryptophan hydroxylase. The level of 5-hydroxytryptophan in blood and tissue is very low. As soon as formed, it is decarboxylated into 5-hydroxytryptamine, also known as serotonin (Höglund et al., 2019).

Melatonin is a hormone that regulates the circadian rhythm and acts as a powerful antioxidant. The pineal gland is the principal site of melatonin synthesis, and its synthesis and secretion are regulated by the hypothalamic suprachiasmatic nucleus in response to the light–dark cycle (Sumsuzzman et al., 2021). TRP is also the primary precursor of melatonin. In the melatonin biosynthetic pathway, serotonin undergoes N-acetylation to form N-acetylserotonin by the action of arylalkylamine N-acetyltransferase, which is subsequently methylated to produce melatonin (Figure 1) (Kim et al., 2022).

#### 1.1.3 Indole pathway

Under physiological conditions, approximately 4–6% of dietary TRP is metabolised by the gut microbiota into indole and other indole-derived metabolites by bacterial enzymes, namely tryptophanase. These metabolites act as key signalling molecules that influence intestinal integrity, immune modulation, and systemic homeostasis. Several gut bacterial species, including *Escherichia coli*, *Clostridium sporogenes*, *Ruminococcus gnavus*, *Lactobacillus* spp., and *Bifidobacterium* spp., participate in TRP metabolism. Among these, the bacterial enzyme tryptophanase catalyses the conversion of TRP into indole, pyruvate, and ammonia. Indole is readily absorbed across the intestinal epithelium and transported to the liver, where it undergoes oxidation and sulfation to form indoxyl sulfate (IS), a protein-bound uremic toxin (PBUT) that is normally excreted through the kidneys. In CKD, impaired renal clearance increases the accumulation of IS and contributes to systemic toxicity (Cheng et al., 2020). In addition to indole, gut microbial metabolism generates several biologically active TRP metabolites, including indole-3-acetic acid (IAA), indole-3-propionic acid (IPA), indole-3-lactic acid (ILA), and tryptamine (Williams et al., 2014). Notably, many indoles and its derivative metabolites, including indole, IS, IAA, and IPA, function as ligands of the aryl hydrocarbon receptor (AhR), a ligand-activated transcription factor involved in immune regulation, epithelial barrier maintenance, and inflammatory signalling (Curran and Kopp, 2022). Under physiological conditions, AhR activation contributes to intestinal and immune homeostasis. However, the balance of indole-derived metabolites is altered in CKD due to gut dysbiosis and impaired renal clearance, which results in the accumulation of potentially toxic substances. One way these metabolites may be associated with inflammation, oxidative stress, endothelial dysfunction, and disease progression is through altered AhR signalling. As a result, there is growing interest in the TRP–indole–AhR axis as a potential link between gut microbial metabolism and disorders related to chronic kidney disease (Figure 1) (Li et al., 2021; Cheng et al., 2020; Williams et al., 2014; Paeslack et al., 2022; Tang et al., 2023).

## 2 Mechanism of cognitive impairment in CKD

Cognitive impairment is common in patients with CKD and is associated with multiple factors. However, genetic factors contribute substantially to cognitive dysfunction in children with CKD. Cerebrovascular injury and the accumulation of uremic toxins are the predominant drivers in adults. Patients with ESRD exhibit a markedly increased burden of cerebral small vessel disease, as evidenced by a higher incidence of stroke, subcortical infarcts, cerebral microbleeds, and white-matter hyperintensities (Bi et al., 2025). These lesions are strongly associated with impaired cognition and are promoted by CKD-related comorbidities, including hyperglycaemia, hypertension, anaemia, electrolyte imbalance, uraemic toxicity, and a pro-thrombotic state. Treatment-related factors, such as dialysis-associated hypotension, aluminium exposure, and polypharmacy, further aggravate cerebrovascular vulnerability (Drew et al., 2019).

The systemic accumulation of uremic toxins due to progressive loss of renal clearance. Uremic toxins are commonly classified by their physicochemical properties into small, water-soluble compounds, middle molecules, and large, protein-bound compounds (Lim et al., 2021). PBUTsare unable to be removed in dialysis. Collectively, growing evidence suggests that uremic toxins represent particularly important contributors to CKD-associated cognitive decline (Franco et al., 2019).

Systemic endothelial dysfunction has been proposed as an important mechanism linking uremic toxin accumulation with cerebrovascular injury. The vascular endothelium plays a pivotal role in regulating vascular tone, permeability, inflammation signalling, thrombosis, and angiogenesis (da Cunha et al., 2020). Patients with CKD, especially those with ESRD undergoing dialysis, exhibit molecular and structural evidence of endothelial dysfunction, including an increase in the wall-to-lumen ratio and upregulation of adhesion and coagulation-related molecules, such as VCAM-1, ICAM-1, ICAM-3, and angiopoietin-2 (Olczyk et al., 2022; Libório et al., 2024). Furthermore, the endothelial glycocalyx is significantly decreased in CKD. However, the exact mechanism of endothelial dysfunction that leads to CI in CKD following exposure to uremic toxins is not clear.

Increasing preclinical studies, supported by clinical observational studies, suggest that uremic toxins may contribute to BBB disruption. Multiple CKD models showed increased BBB permeability, which correlated with cognitive decline and elevated levels of PBUTs (Bobot, 2023). Exposure to trimethylamine-N-oxide further suppresses the expression of tight junction proteins in the animal model (Janeiro et al., 2023). The coexistence of elevated uremic toxins and BBB dysfunction may facilitate the entry of neurotoxic compounds and inflammatory mediators into the brain. Impaired BBB may promote neuroinflammatory signalling and microglia activation, which release cytokines, increase ROS and lipid mediators, disrupt synaptic function and neuronal survival (da Fonseca et al., 2014). In addition, CKD is also associated with vascular injury, driven by atherosclerosis, which further compromises cerebral perfusion. They are particularly susceptible to hemodynamic stress imposed by hypertension, diabetes, and obesity, providing a mechanistic explanation for the substantial vascular overlap between renal and cerebral disease in CKD.

**Figure 2:**
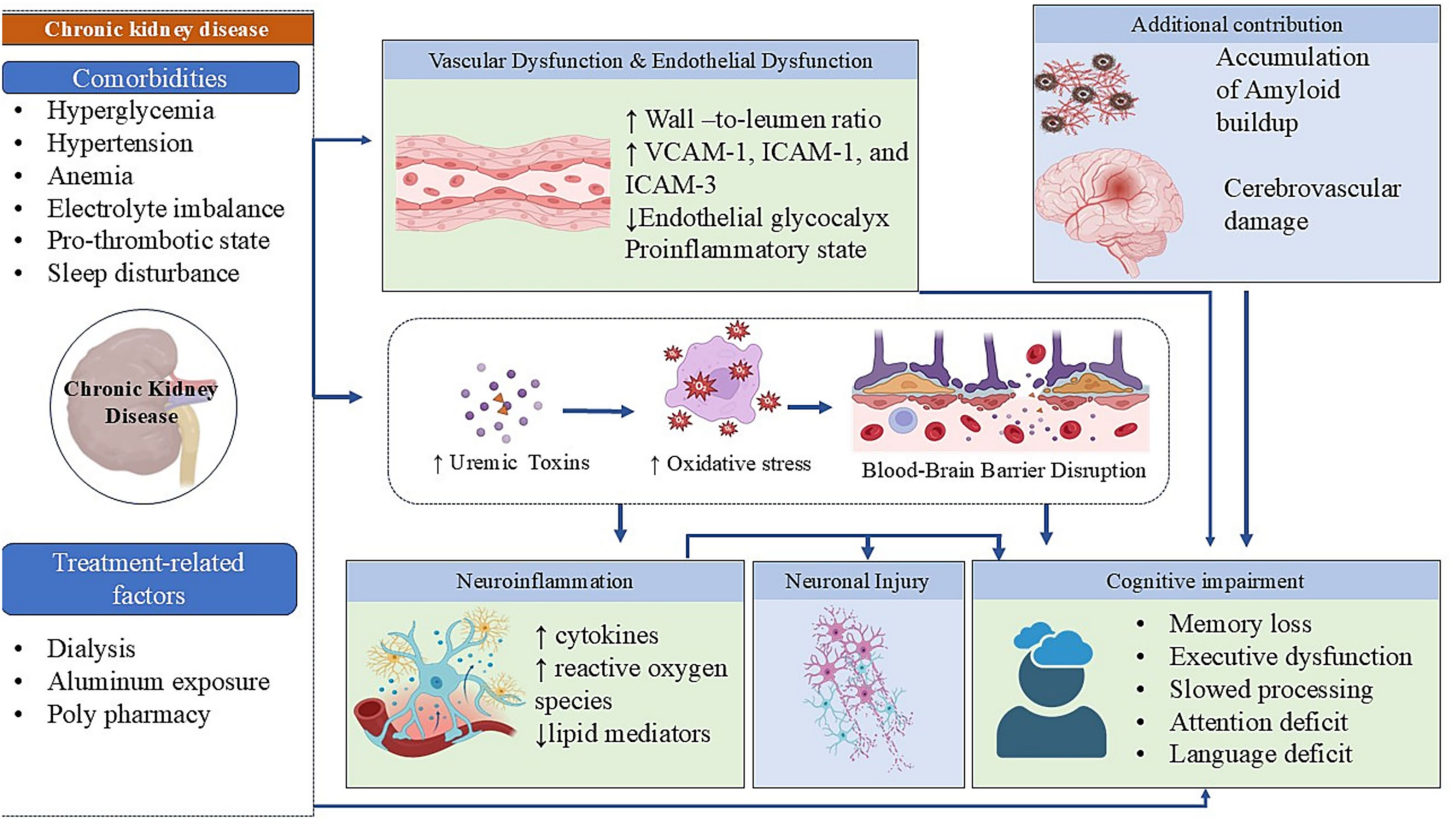
Mechanism linking chronic kidney disease (CKD) and cognitive impairment. CKD-associated comorbidities such as hyperglycaemia, hypertension, anaemia, electrolyte imbalance, and pro-thrombotic state, together with treatment-related factors such as dialysis, aluminium exposure, and polypharmacy, can directly contribute to cognitive impairments. Decreased renal clearance leads to increased accumulation of uremic toxins, which increases oxidative stress and contributes to blood–brain barrier disruption, facilitating neurotoxic exposure, neuroinflammation, and neuronal injury. Comorbidities and uremic toxins contribute to vascular dysfunction and endothelial injury. Additionally, amyloid accumulation and cerebrovascular damage may further aggravate brain injury. Collectively, interrelated pathways contribute to cognitive impairment.

Acute CI may be influenced by oxidative stress, electrolyte imbalance, acid–base imbalance, and the direct neurotoxic effect of accumulated solutes. In contrast, chronic CI is driven by vascular damage, endothelial dysfunction, and nutritional impairment, with amyloid deposition contributing to long-term cognitive decline (Imenez Silva et al., 2021; Yan et al., 2024). Sleep disturbance is very common in CKD, worsening brain injury by impairing glymphatic clearance. Sleep disturbance is also linked to dysregulation of the serotonin-melatonin pathway in CKD, which may contribute to circadian rhythm disruption and impaired sleep quality. Alteration in this pathway affects cognitive outcomes in CKD, given the known link between sleep disorders and CI. However, sleep disturbance reduces the glymphatic clearance of amyloid-β (Aβ) and other neurotoxic metabolites, leading to their accumulation (Figure 2) (Merlino et al., 2025). Our previous study demonstrated that CI in CKD patients is associated with increased accumulation of Aβ and phosphorylated tau (pTau) proteins. Although these biomarkers overlap with those observed in Alzheimer’s disease, the presence of similar biomarker profiles does not necessarily indicate shared underlying disease mechanisms, and the relationship between CKD-associated CI and Alzheimer’s disease pathology remains incompletely understood (Ganesan et al., 2021).

### 2.1 Dysregulation of tryptophan metabolism in cognitive impairment in chronic kidney disease

#### 2.1.1 Dysregulation of kynurenine metabolites in chronic kidney disease leads to cognitive impairment

Substantial evidence that the KP is markedly dysregulated in CKD, and this metabolic shift is increasingly linked to the development of CI. Systemic inflammatory activation is a primary driver of this metabolic redirection. Pro-inflammatory cytokines, particularly tumour necrosis factor-*α* and interleukin-6, strongly activate indoleamine-2,3-dioxygenase-1 (IDO-1), resulting in the accelerated conversion of TRP to KYN and a higher KYN/TRP ratio. Clinical studies have shown that circulating TRP levels are associated with a worsening of estimated glomerular filtration rate (eGFR). However, KYN and its downstream metabolites, including KYN, 3-OH KYN, and QA, were markedly increased and positively correlated with the inflammatory marker neopterin (Debnath et al., 2017).

Experimental evidence provides additional mechanistic insights into these findings. Metabolomic profiling of the kidney cortex and brain tissue in CKD animal models have demonstrated the accumulation of downstream KYN pathway metabolites, including KYN, 3-OH KYN, and QA, together with the upregulation of inflammatory mediators (Saliba et al., 2025). These findings suggest that dysregulating kynurenine metabolism may be associated with neuroinflammation processes relevant to CI, although direct causal relationships remain incompletely established.

**Figure 3:**
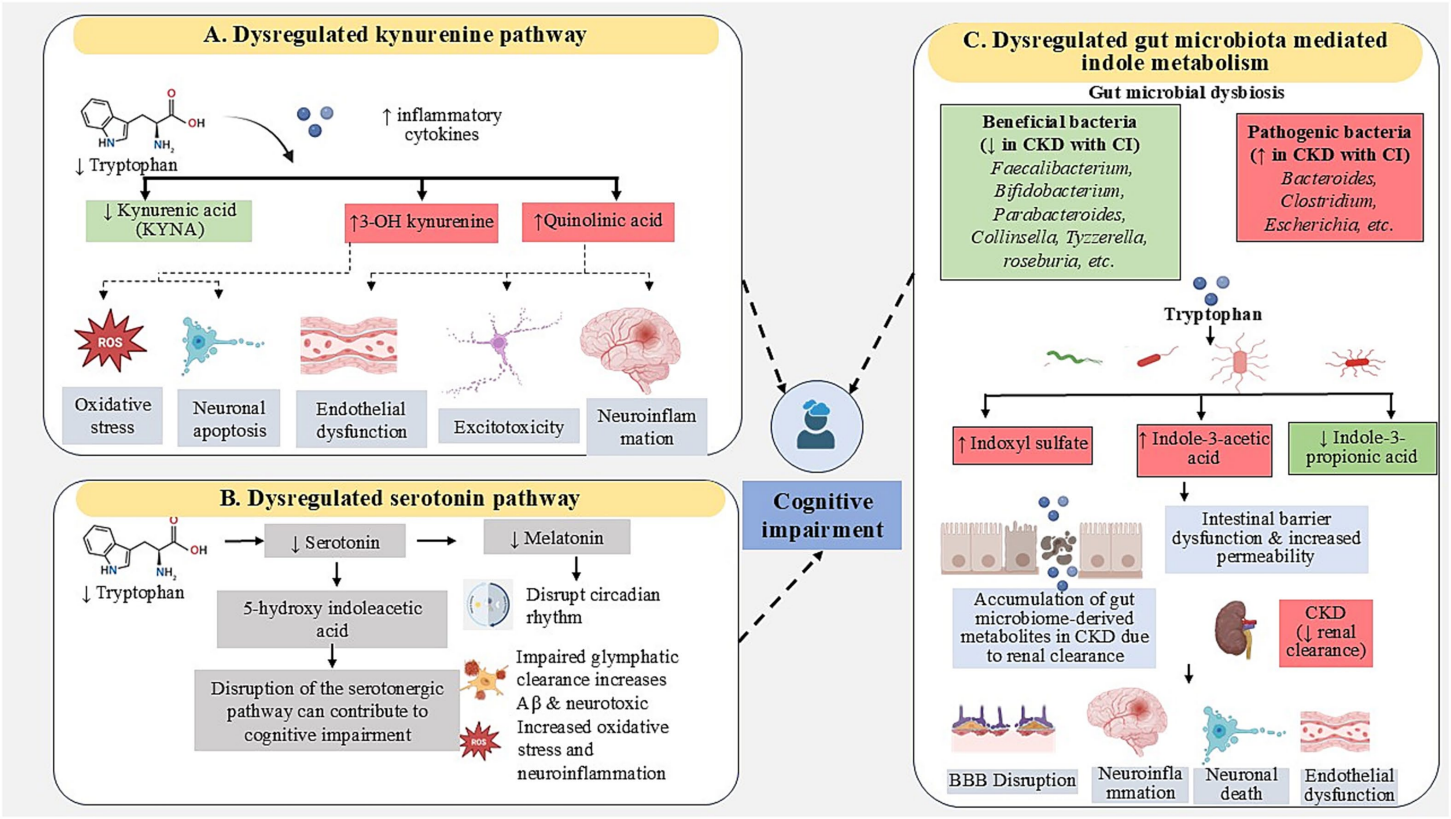
Interconnected dysregulation of tryptophan metabolism drives cognitive impairment in chronic kidney disease. **(A)** In CKD, signalling from inflammation increases tryptophan’s diversion into the kynurenine pathway, which lowers neuroprotective kynurenic acid (KYNA) and raises neurotoxic metabolites such as quinolinic acid and 3-hydroxykynurenine. Dysregulation promotes Oxidative stress, neuronal death, endothelial dysfunction, excitotoxicity, and neuroinflammation, which are all encouraged by this imbalance; **(B)** Gut dysbiosis further alters tryptophan metabolism, increasing harmful indole-derived toxins and reducing beneficial metabolites. These changes weaken the intestinal barrier, allow toxin accumulation due to reduced kidney clearance, and trigger blood–brain barrier disruption, neuroinflammation, and neuronal injury; **(C)** Reduced tryptophan availability decreases serotonin and melatonin synthesis, alters 5-hydroxyindoleacetic acid levels, disrupts circadian regulation, impairs glymphatic clearance of neurotoxic proteins, and exacerbates oxidative stress and neuroinflammation, contributing to cognitive dysfunction.

Under physiological conditions, peripheral QA is largely excluded from the brain’s NAD + production. However, in CKD, sustained systemic inflammation increases the expression of IDO-1, KMO, and Kynureninase in immune cells or brain-resident microglia, favoring the neurotoxic branch and promoting excessive local QA synthesis (Karu et al., 2016). Experimental studies suggest that elevated QA can act as a potent agonist of the N-methyl-D-aspartate receptor (NMDA) and the α7 nicotinic acetylcholine receptor, inducing excitotoxic neuronal damage. In addition, QA has also been associated with oxidative stress, which disrupts glutamate reuptake by astrocytes and increases inflammatory signalling (Figure 3) (Tavares et al., 2002; Kang et al., 2020). However, the extent to which these mechanisms contribute directly to CI in human CKD remains incompletely understood.

Beyond QA, several additional kynurenine metabolites contribute to neuronal and vascular impairment in CKD. Clinical studies have reported an association between KYN metabolites and endothelial dysfunction, which may contribute to CI by affecting cerebral vascular integrity (Pawlak et al., 2010). Experimental studies have detected 3-HK and KYN in multiple brain regions in experimental CKD models, where these metabolites have been associated with oxidative damage, mitochondrial dysfunction, and apoptotic signalling (Topczewska-Bruns et al., 2002).

Although KYNA is generally considered a neuroprotective metabolite due to its antagonistic action on the NMDA receptors and α7 nicotinic acetylcholine receptors, excessive accumulation of KYNA may impair the synaptic transmission and cognitive process by reducing glutamatergic and cholinergic signalling (Albuquerque and Schwarcz, 2013). Therefore, altered KYNA concentration should not necessarily be interpreted as beneficial, and functional consequences may depend on the balance between neuroprotective and neurocognitive effects. Overall, cognitive dysfunction in CKD is likely influenced by an imbalance between neuroprotective and neurotoxic kynurenine metabolites rather than by a single metabolite alone.

#### 2.1.2 Dysregulation of indole metabolites in chronic kidney disease with cognitive impairment

Beyond the KYN pathway, indole and its downstream derivatives constitute an additional and increasingly important branch of TRP metabolism in CI in CKD. The gut microbiota produces these indole derivatives and has emerged as a potential mediator linking gut dysbiosis, systemic inflammation and CI in CKD. Among these compounds, IAA and IS are our major tryptophan-derived uremic toxins produced by microbes. Under physiological conditions, these toxins are eliminated predominantly by tubular secretion via organic anion transporters or organic cation transporters (OCT) for the removal of PBUTs (Masereeuw et al., 2014). Clinical microbiome studies indicate that CKD patients with mild cognitive impairment exhibit a distinct dysbiosis signature compared with cognitively preserved individuals. Reductions in several commensal taxa, including members of the class *Coriobacteriia* and the genera *Tyzzerella, Blautia, Lachnospira*, and *Roseburia*, as well as families such as *Sphingomonadaceae* and *Xanthomonadaceae*, have been consistently reported (Zhu et al., 2020). Importantly, beneficial bacteria such as *Faecalibacterium, Megamonas, Bifidobacterium, Parabacteroides, Collinsella, Phascolarctobacterium,* and *Tyzzerella* are positively associated with cognitive performance in patients receiving haemodialysis (Gao et al., 2024). Whereas potentially pathogenic genera, including *Bacteroides* and *Clostridium*, are enriched in CKD (Nallu et al., 2017).

Several gut bacteria, particularly species within *the Clostridia, Bacteroides, and Escherichia genera*, produce IAA from tryptophan. Among these, *Bacteroides thetiotaomicron* has emerged as a major contributor to circulating IAA levels and is regarded as a key symbiont stabilising colonic microbial ecology. Dysbiosis-driven alterations in these microbial communities, therefore, directly modulate indole and IAA (Wu et al., 2024).

Under physiological conditions, circulating indole and its derivatives is very low concentrations. CKD is associated with disruption of the intestinal epithelial barrier, increased gut permeability and translocation of microbial products. This promotes systemic inflammation and facilitates the entry of gut-derived indole metabolites into the circulation, where they contribute to the uremic toxicity. Several experimental studies have demonstrated the neuronal toxicity in a neuronal cell model and increased the number of pyknotic neurons in the hippocampus of adenine-induced CKD models, in addition to elevated oxidative stress markers. However, the experimental studies suggest that IS may disrupt BBB integrity through activation of the aryl hydrocarbon receptor (AhR), thereby facilitating further influx of circulating neurotoxic solutes and aggravating cerebral injury (Watanabe et al., 2021).

Neuroinflammation is another major hallmark of CI. At the cellular level, experimental evidence indicates that IS can activate the AhR and NF-κB signalling pathways in astrocytes, glial cells, and primary neurons, leading to neuroinflammatory responses (Adesso et al., 2017). IS also induces astrocyte apoptosis through inhibition of the MAP-kinase pathway, including extracellular signal-regulated kinase. MAPK/ERK kinase, c-Jun N-terminal kinase and p38, and inhibits glycolytic metabolism in astrocytes (Jeong et al., 2024). In neuronal models, IS triggers oxidative stress, alters the Bax/Bcl-2 ratio, activates the FAS/FAS-ligand axis, and increases cleaved caspase-3 levels, indicating early extrinsic apoptotic signalling. Collectively, these findings suggest that IS may contribute to neurotoxicity and neuronal dysfunction in CKD; however, the extent to which these experimental observations translate into CI remains to be fully established (Hsieh et al., 2025).

Clinical studies further support the relevance of indole metabolites to cognitive outcome. Circulating IS, Indole, and IAA are increased in CKD patients with mild cognitive impairment. In hemodialysis cohorts, plasma IAA levels have been reported to correlate inversely with cognitive performance (Lin et al., 2019). Despite these associations, the specific gut-kidney-brain axis mechanism linking IAA to neurocognitive decline remains poorly defined and further investigation is required.

The role of IS in cardiovascular disease among patients with CKD has been widely studied. Similarly, IS exerts an indirect effect on the cerebral vasculature, inducing endothelial dysfunction through inflammation, oxidative stress, and a pro-coagulant phenotype (Cho et al., 2025). Since AhR is extensively expressed in the CNS, inappropriate activation of AhR may be a factor in the CI and poor neurovascular function in CKD (Bobot et al., 2020).

In contrast, indole-3-propionic acid (IPA) has emerged as a potentially protective microbial metabolite. IPA, produced predominantly by *Clostridium sporogenes* and relevant bacterial species, is significantly reduced in diabetic kidney disease and shows a positive association with eGFR. However, the contributions of IPA and IPA-producing bacteria to cognitive outcomes in CKD have not yet been systematically evaluated. Extensive experimental evidence supports the neuroprotective properties of IPA, indicating that loss of this metabolite may further predispose CKD patients to neurological vulnerability, even though the contribution of IPA-producing bacteria to cognitive outcomes in CKD has not yet been systematically evaluated. While alterations in gut microbial composition have been associated with cognitive dysfunction in CKD, evidence linking individual bacterial taxa to specific neurological or cognitive outcomes remains limited. Most studies involve relatively small cohorts and report associative rather than causal relationships. Consequently, the contribution of individual microbial species to CKD-associated cognitive impairment, as well as their interactions with tryptophan metabolism, requires further validation in larger and more diverse patient populations (Figure 3) (Zeng et al., 2025).

#### 2.1.3 Dysregulation of the serotonin-melatonin pathway in cognitive impairment among CKD

The serotonin and melatonin pathway is a major branch of tryptophan metabolism that plays an essential role in regulating mood, cognition, circadian rhythm, and sleep–wake homeostasis. In chronic inflammation, activation of KP diverts TRP away from serotonin synthesis, resulting in an imbalance of neuroactive metabolites.

Serotonin is a key neurotransmitter involved in the regulation of mood, learning, memory, and cognitive function. Clinical investigation has shown that patients undergoing hemodialysis have lower levels of circulating serotonin, which may indicate altered serotonergic neurotransmission in advanced CKD. A major limitation of using serotonin as a biomarker is that it quickly breaks down into 5-hydroxyindoleacetic acid (5-HIAA) (Jayamohananan et al., 2019). Notably, the 5-HIAA CSF level in CKD is not yet quantified. But the plasma level of 5-HIAA has been reported to increase ninefold in CI among CKD patients (Figure 3) (Schefold et al., 2009). However, it remains unclear whether this increase primarily reflects reduced renal clearance or enhanced serotonin turnover. Consequently, additional studies are required to understand the relationship between serotonergic dysfunction and cognitive decline in CKD. Melatonin, a downstream metabolite of serotonin, is a central regulator of circadian rhythm and sleep wake homeostasis. Melatonin metabolism is markedly dysregulated in CKD, with consistently reduced nocturnal melatonin secretion observed in patients with ESRD. This abnormality persists despite renal replacement therapies, including renal transplantation and haemodialysis, indicating the sustained disruption of the circadian rhythm (Maung et al., 2016). Reduced melatonin availability contributes to the high prevalence of sleep disturbances in CKD and may increase susceptibility to cognitive dysfunction. In addition, impaired sleep has been implicated in reduced glymphatic clearance of amyloid-β and other neurotoxic metabolites, thereby promoting their accumulation in the brain. Diminished melatonin signalling may also exacerbate oxidative stress and neuroinflammation, further contributing to neuronal injury and cognitive decline (Figure 3) (Li et al., 2025).

A recent randomized clinical trial involving 102 haemodialysis patients demonstrated that administration of melatonin (3 mg nightly for 6 weeks) significantly improved sleep quality and cognitive function measured by the Pittsburgh Sleep Quality Index (PSQI) and Montreal Cognitive Assessment (MoCA), respectively, compared with the placebo group. However, studies have various limitations, including relatively small sample size, short duration, single-centre design, and reliance on a global cognitive screening tool rather than comprehensive neuropsychological assessment. Findings suggest melatonin supplementation may have therapeutic potential for CI in CKD, but long-term effects and neuroprotective actions remain uncertain. Larger, multicentre clinical trials with extended follow-up and standardized cognitive endpoints are necessary to confirm the efficacy and safety of melatonin-based therapies (Jafari et al., 2023).

Collectively, current evidence suggests that dysregulation of the serotonin-melatonin pathway contributes to CI in CKD through multiple mechanisms, including oxidative stress, neuroinflammation, sleep disturbance, circadian rhythm disruption, and decreased neurotransmission. However, more research is needed to understand the relative interaction with the kynurenine pathway fully, remain incompletely understood and requires further investigation (Table 1).

**Table 1:** Dysregulated tryptophan metabolism in chronic kidney disease: causes and cognitive impairment mechanisms.

Metabolite	Alteration in CKD	Evidences	Reason behind dysregulation of tryptophan metabolism	Mechanism implicated in cognitive impairment	References
Kynurenine	Increased	Clinical association and experimental	IDO1 is activated by and pro-inflammatory cytokines (TNF-*α*, IL-6), which increases the KYN/TRP ratio and TRP breakdown.	may promote neurotoxic kynurenine flux, endothelial dysfunction, and inflammation-associated cognitive injury	Debnath et al. (2017); Karu et al. (2016)
3-Hydroxykynurenine	Increased	Experimental study	Increased flux through the neurotoxic branch and elevated KMO activity.	In vitro and *in vivo* studies suggest that elevated 3-HK may contribute to oxidative stress, mitochondrial dysfunction, and neuronal apoptosis	Saliba et al. (2025); Topczewska-Bruns et al. (2002)
Quinolinic acid	Increased	Clinical association and experimental	Increased downstream metabolism during inflammation via the neurotoxic kynurenine branch	Acts as an agonist of the N-methyl-D-aspartate receptor (NMDA) and the α7 nicotinic acetylcholine receptor, may induce excitotoxic neuronal damage	Kang et al. (2020); Karu et al. (2016); Tavares et al. (2002)
Kynurenic acid	Decreased	Experimental; conflicting clinical evidence	Disturbed balance between KAT and KMO pathways	In some conditions, decreases in KYNA reduce the neuroprotective effect, excessive synaptic inhibition affecting cognition.	Topczewska-Bruns et al. (2002)
Indoxyl sulfate	Increased	Clinical association and experimental	Liver sulfation, reduced renal clearance, gut dysbiosis, and increased microbial indole synthesis	Experimental studies suggest that IS is associated with BBB disruption, AhR activation, neuroinflammation, and endothelial dysfunction	Adesso et al. (2017); Jeong et al. (2024); Watanabe et al. (2021)
Indole-3-acetic acid	Increased	Clinical association and experimental	Overproduction brought on by dysbiosis and decreased renal clearance	Elevated IAA is associated with Neurovascular dysfunction, which is associated with impaired cognition	Masereeuw et al. (2014); Watanabe et al., 2021
Indole-3-propionic acid	Decreased	Experimental study	Beneficial IPA-producing microbiota loss in CKD dysbiosis.	Loss of IPA may reduce antioxidant/neuroprotective effects, increase vulnerability to neurological injury	Zeng et al. (2025)
Serotonin	Decreased	Clinical study	Tryptophan was redirected to the indole and kynurenine pathways.	Reduced serotonergic signalling affects cognition and behaviour	Jayamohananan et al. (2019); Schefold et al. (2009)
5-Hydroxyindoleacetic acid	Increased	Clinical study	Reduced renal clearance or altered serotonin turnover	Altered 5-HIAA levels may reflect disrupted neurotransmitter homeostasis and have been associated with cognitive vulnerability.	Jayamohananan et al. (2019); Schefold et al. (2009)
Melatonin	Decreased	Clinical study	Impaired circadian regulation and reduced melatonin secretion in CKD	Melatonin deficiency may contribute to sleep disruption, impaired glymphatic clearance, and oxidative stress, potentially influencing cognitive function.	Maung et al. (2016)

## 3 Diagnostic and therapeutic approach of cognitive impairment in chronic kidney disease

### 3.1 Diagnostic approach to cognitive impairment in chronic kidney disease

A detailed and multifaceted strategy is necessary for the proper screening, diagnosis and prognosis of CI in patients with CKD. Clinical evaluation should consider the stage and duration of CKD, the adequacy of renal replacement therapy, particularly in patients undergoing hemodialysis, the treatment course, current medication, dietary pattern, and severity of comorbidities such as Diabetes mellitus and systemic hypertension. These factors influence cognitive performance and may confound clinical interpretation.

Currently, Cognitive screening in CKD is commonly performed using standardised questionnaire tools, such as the Mini-Mental State Examination (MMSE) and MoCA (Amatneeks and Hamdan, 2019). However, these approaches have limited sensitivity for early detection and fail to capture the metabolic mechanisms underlying cognitive decline (Tiffin-Richards et al., 2014; Wang K. et al., 2025). This has driven interest in tryptophan-derived metabolites as potential diagnostic and prognostic biomarkers.

Emerging evidence suggests that dysregulation of tryptophan metabolism is associated with CI in CKD and may provide a mechanistic basis for developing a novel biomarker. Most studies have quantified peripheral blood tryptophan metabolites using ultra-high-performance liquid chromatography- mass spectrometry (UHPLC–MS), a highly sensitive and specific technique that simultaneously detects multiple metabolites. However, several methodologies and biological factors affect the interpretation of these findings (Zakrocka et al., 2026). Circulating metabolite concentration may be influenced by factors such as the time of sample collection, sample preparation, measurement methodology, dietary intake, gut microbiota composition, systemic inflammation, CKD stage, dialysis modality, medications, and residual renal function, thereby affecting reproducibility and inter-study variability (Lazarevic et al., 2024). Furthermore, although most studies rely on plasma or serum measurements, it remains uncertain whether peripheral metabolite concentrations accurately reflect levels within the central nervous system (CNS). While KYN readily crosses the BBB, several downstream metabolites, including QA and KYNA, exhibit limited transport and may also be synthesised locally within the CNS. Cerebrospinal fluid (CSF) analysis may provide a more direct assessment of central tryptophan metabolism; however, CSF-based studies in CKD are lacking. Consequently, larger longitudinal studies with standardised analytical approaches are needed to establish the sensitivity, specificity, and predictive value of tryptophan-derived metabolites as diagnostic and prognostic biomarkers of CKD-associated CI.

In future diagnostic strategies, benefit from integrating tryptophan metabolite profiling with standardised cognitive assessment (MoCA), neuroimaging, and longitudinal clinical evaluation to improve early diagnosis, risk stratification, and prognostic assessment in CKD-associated CI. Multimodal approaches enhance diagnostic accuracy and provide additional information on metabolic, structural, and functional changes associated with CI in CKD.

### 3.2 Treatment strategies targeting tryptophan-related pathways to mitigate cognitive impairment in CKD

Major Therapeutic Strategies aimed at addressing the CI in CKD include modulation of gut microbiota using probiotics, reduction of uremic toxin burden through intestinal adsorbents such as AST 120 (kremezin), and restoration of circadian regulation with melatonin.

The gut-brain-kidney axis may be modulated by probiotics, which are live bacteria that provide health advantages when taken in sufficient quantities. Probiotics have been shown to lower plasma levels of gut-derived uremic toxins in CKD animal models (Ranganathan et al., 2005). These results are further supported by clinical evidence; a 12-week randomised controlled trial in patients receiving hemodialysis found that supplements containing *Lactobacillus acidophilus*, *Lactobacillus casei*, and *Bifidobacterium bifidum*, or a placebo, significantly decreased plasma glucose, oxidative stress markers, and inflammatory markers while increasing antioxidant capacity (Soleimani et al., 2017). Furthermore, probiotics have been linked to improvements in cognitive performance in a several disease states, suggesting a neuroprotective role (Ma et al., 2025). However, evidence directly evaluating cognitive outcomes in CKD remains limited, and further validation is required.

AST 120 (Kremezin) is an oral intestinal carbonaceous adsorbent, representing another therapeutic strategy targeting uremic toxin accumulation. By adsorbing gut-derived indole compounds such as indoxyl sulfate, it reduces systemic toxins burden (Schulman et al., 2014). Preclinical and limited clinical studies suggest that AST-120 may improve cognitive and behavioural outcomes in CKD, possibly through reduction of indoxyl sulfate levels and attenuation of neuroinflammation (Yu et al., 2024). After 48 weeks of treatment, a randomised controlled trial found improvements in cognitive function (Cha et al., 2022). However, the interpretability of these results is limited due to the lack of standardised cognitive assessment instruments. Although meta-analyses have shown improvements in renal and metabolic parameters, there is still insufficient solid clinical data to establish its benefits for cognition (Altunkaynak et al., 2024).

Melatonin, a key regulator of circadian rhythm and a potent antioxidant, has also shown therapeutic potential in CKD-associated CI. Clinical studies suggest that melatonin supplementation may improve sleep quality and short-term cognitive function in patients undergoing dialysis. Nevertheless, the available evidence is limited by relatively small sample sizes, short follow-up periods, and heterogeneous cognitive endpoints. Therefore, larger controlled trials are required to establish the long-term efficacy and safety of melatonin supplementation in CKD-associated cognitive impairment (Jafari et al., 2023).

Therapeutic targets within the kynurenine pathway primarily include the rate-limiting enzymes IDO1 and TDO2. Several IDO1 inhibitors, including epacadostat, linrodostat, navoximod, and indoximod, have advanced to clinical trials, mostly in oncology, where they have demonstrated the feasibility of modulating tryptophan metabolism in humans (Kang et al., 2025). Although clinical studies evaluating IDO1 inhibitors in CKD or CKD-associated cognitive impairment are currently lacking, suppression of excessive activation of the kynurenine pathway may reduce neuroinflammation, oxidative stress, and the accumulation of neurotoxic metabolites. Collectively, these findings support further investigation of IDO1 and KMO inhibition as potential therapeutic strategies for CKD-associated cognitive impairment.

For CKD-related cognitive impairment, regulation of tryptophan metabolism offers an effective approach to personalised medicine. Patients with CKD show substantial heterogeneity in gut microbiota composition, inflammatory status, renal function, and circulating tryptophan metabolite profiles, all of which may influence disease progression and therapeutic responses. Metabolomic and gut microbiome profiling have enabled the identification of altered kynurenine pathway activity, reduced serotonin and melatonin levels, and elevated indole-derived uremic toxins, which serve as biomarkers for patient stratification. These approaches may facilitate personalised therapies based on individual metabolic signatures. Microbiota-targeted interventions or enzyme inhibitors, such as IDO1 and KMO inhibitors, could be selected according to the predominant metabolic disturbance. Although still investigational, integrating microbiome and metabolome profiling into clinical practice may enhance risk prediction and optimise treatment strategies for CKD-associated cognitive impairment.

## 4 Conclusion and future direction

CKD is increasingly recognised as a systemic disorder with multiple consequences, among which cognitive impairment represents a major yet underappreciated complication. Emerging evidence suggests that dysregulated tryptophan metabolism, encompassing the kynurenine, indole, and serotonin pathway may represent an important mechanistic pathway linking renal impairment, systemic inflammation, and neurocognitive decline. Dysregulation of these pathways has been implicated in several mechanisms associated with cognitive impairment, including neuroinflammation, oxidative stress, excitotoxicity, endothelial dysfunction, and blood–brain barrier disruption, thereby supporting the concept of a gut–kidney–brain axis in CKD. Furthermore, tryptophan-derived metabolites have emerged as promising biomarkers and potential therapeutic targets. From a clinical perspective, current diagnostic approaches remain largely dependent on neuropsychological testing and conventional laboratory parameters, with limited availability of reliable and specific biomarkers. Although tryptophan-derived metabolites show strong mechanistic relevance and emerging diagnostic potential, their clinical applicability is still constrained by insufficient validation, variability across patients’ population, and overlap with other CKD-related complications. Therefore, integrating metabolomic profiling with clinical assessments, neuroimaging, and biochemical markers represents a promising strategy for improving early detection and risk stratification.

Future research should prioritize large-scale, longitudinal, and multicenter human studies to clarify the temporal relationship between tryptophan metabolites and cognitive decline, and to validate these metabolites as diagnostic and prognostic biomarkers. Such studies should incorporate receiver operating characteristic (ROC) analysis and other performance metrics to determine the sensitivity, specificity, and predictive utility of candidate biomarkers. A deeper understanding of the gut microbiota and its role in modulating indole metabolism may open new avenues for targeted therapeutic interventions, including microbiome-based strategies such as probiotics, prebiotics, and dietary modulation. Therapeutic targeting of specific metabolic pathways, particularly inhibition of IDO-1, KMO, and the neurotoxic kynurenine branch, or restoration of melatonin signalling, holds potential but requires rigorous clinical evaluation to establish efficacy, safety, and long-term outcomes. Future intervention trials should include cognitive outcomes as primary endpoints to determine whether modulation of tryptophan metabolism can meaningfully improve cognitive function in CKD.

Additionally, future studies should also concentrate on personalised treatment strategies that take individual metabolic profiles, CKD stage, comorbidities, and dialysis method into consideration. Integration of advanced multi-omics approaches, including metabolomics, transcriptomics, and gut microbiome analysis, may further elucidate disease mechanisms and identify novel therapeutic targets. Overall, improving cognitive outcomes and quality of life in patients with CKD will require an integrative approach that combines mechanistic insights with clinical translation and personalised therapeutic strategies.

## Glossary

3-HAA: 3-Hydroxyanthranilic Acid
3-HK: 3-Hydroxykynurenine
3-HAO: 3-Hydroxyanthranilate 3,4-dioxygenase
5-HIAA: 5-Hydroxyindoleacetic Acid
5-HT: 5-Hydroxytryptamine (Serotonin)
AA: Anthranilic Acid
AANAT: Arylalkylamine N-acetyltransferase
ACMS: 2-Amino-3-carboxymuconate-6-semialdehyde
AhR: Aryl Hydrocarbon Receptor
ASMT (HIOMT): Acetylserotonin O-methyltransferase
BBB: Blood–Brain Barrier
CI: Cognitive Impairment
CKD: chronic kidney disease
CNS: Central Nervous System
eGFR: Estimated Glomerular Filtration Rate
ERK: Extracellular Signal-Regulated Kinase
ESRD: End-Stage Renal Disease
fldAIBC: Gene cluster for indole-3-propionate pathway
HD: Hemodialysis
IAA: Indole-3-acetic Acid
IA: Indole-3-acrylate
IAId: Indole-3-acetaldehyde
IAM: Indole-3-acetamide
IDO1: Indoleamine 2,3-dioxygenase 1
IDO2: Indoleamine 2,3-dioxygenase 2
IFN-*γ*: Interferon-gamma
IL-1β: Interleukin-1 beta
IL-6: Interleukin-6
IPA: Indole-3-propionic Acid
IpyA: Indole-3-pyruvate
IS: Indoxyl Sulfate
JAK–STAT: Janus Kinase–Signal Transducer and Activator of Transcription
JNK: c-Jun N-terminal Kinase
KAT: Kynurenine Aminotransferase
KMO: Kynurenine 3-monooxygenase
KP: Kynurenine Pathway
KYN: Kynurenine
KYNA: Kynurenic Acid
KYNU: Kynureninase
MAPK: Mitogen-Activated Protein Kinase
NAD: Nicotinamide Adenine Dinucleotide
NF-κB: Nuclear Factor kappa B
OATs: Organic Anion Transporters
OCT: Organic Cation Transporters
PBUTs: Protein-Bound Uremic Toxins
QPRT: Quinolinate Phosphoribosyltransferase
ROS: Reactive Oxygen Species
TDC: Tryptophan Decarboxylase
TDO: Tryptophan 2,3-dioxygenase
TNF-*α*: Tumor Necrosis Factor-alpha
TnaA: Tryptophanase
TnaB: Tryptophan transporter
TPH: Tryptophan Hydroxylase
TRP: Tryptophan
VCAM-1: Vascular Cell Adhesion Molecule-1
XA: Xanthurenic Acid
